# Feature selectivity can explain mismatch signals in mouse visual cortex

**DOI:** 10.1016/j.celrep.2021.109772

**Published:** 2021-10-05

**Authors:** Tomaso Muzzu, Aman B. Saleem

**Affiliations:** 1 UCL Institute of Behavioural Neuroscience, Department of Experimental Psychology, University College London, 26 Bedford Way, London WC1H 0AP, UK

**Keywords:** predictive coding, sensorimotor, locomotion, active sensing, visual cortex

## Abstract

Sensory experience often depends on one’s own actions, including self-motion. Theories of predictive coding postulate that actions are regulated by calculating prediction error, which is the difference between sensory experience and expectation based on self-generated actions. Signals consistent with prediction error have been reported in the mouse visual cortex (V1) when visual flow coupled to running was unexpectedly stopped. Here, we show that such signals can be elicited by visual stimuli uncoupled to an animal running. We record V1 neurons while presenting drifting gratings that unexpectedly stop. We find strong responses to visual perturbations, which are enhanced during running. Perturbation responses are strongest in the preferred orientation of individual neurons, and perturbation-responsive neurons are more likely to prefer slow visual speeds. Our results indicate that prediction error signals can be explained by the convergence of known motor and sensory signals, providing a purely sensory and motor explanation for purported mismatch signals.

## Highlights

• Mouse primary visual cortex neurons respond to perturbations of visual flow
• Perturbation responses are enhanced by running
• Perturbation responses are stronger in the preferred orientation of the neurons
• Perturbation-responsive neurons are tuned to slow visual speeds

## Graphical abstract

**Figure:**
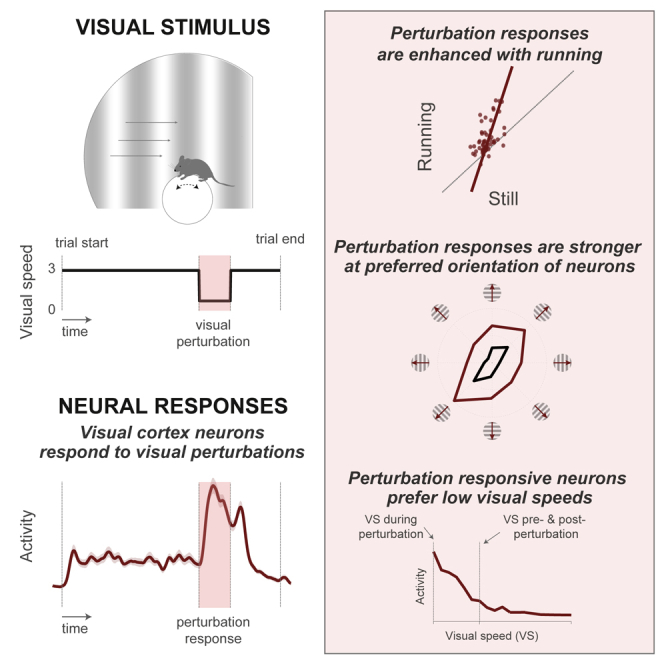

## Teaser

Muzzu and Saleem find neurons in the mouse visual cortex responding to stops or perturbations of open-loop visual flow. Perturbation responses are enhanced by running, and responsive neurons prefer low visual speeds. These results suggest concurrent selectivity for low visual speeds and running can explain sensorimotor mismatch signals in the visual cortex.

## Introduction

Sensation and action are two intertwined processes that the brain continuously executes and adjusts (Claxton, 1975; Wolpert et al., 1995; Rao and Ballard, 1999; Koster-Hale and Saxe, 2013; Friston, 2018). Theories of predictive coding postulate that sensation is an active process that uses information about one’s own actions to distinguish between self-generated and external sensory stimuli. One feature of such predictive coding is the computation of prediction error—the difference between observed features and those expected based on one’s own actions. Prediction error signals have been shown to be encoded in many neural circuits, most famously in the reward system (Schultz et al., 1997), and also in motor (Wolpert and Ghahramani, 2000) and sensory brain regions (Schneider et al., 2014; Rummell et al., 2016).

Locomotion through a familiar environment can generate predictable sensory experiences, including visual flow, that are in turn important in guiding behavior. It has been suggested that errors in the prediction of visual flow are encoded as early as in the primary visual cortex (Keller et al., 2012; Keller and Mrsic-Flogel, 2018), based on large responses to sudden stops of visual flow that were normally coupled to an animal running. The same visual perturbation (cessation of visual flow) played back to a stationary animal elicited smaller responses. In agreement with theories of predictive coding, such activity would provide the mouse visual cortex (V1) with the ability to encode the error between the actual sensory feedback and the expected one, i.e., a visuomotor mismatch signal (Keller et al., 2012). It has been suggested that this visuomotor mismatch is driven by the comparison of excitatory, motor inputs with inhibitory, visual flow inputs (Attinger et al., 2017; Jordan and Keller, 2020). However, neurons in V1 have a wide range of visual speed (or temporal frequency) preferences (Niell and Stryker, 2008; Andermann et al., 2011; Marshel et al., 2011), and running both drives V1 activity and modulates responses to visual stimuli (Niell and Stryker, 2010; Keller et al., 2012; Saleem et al., 2013; Fu et al., 2014; Lee et al., 2014; Busse et al., 2017; Millman et al., 2020). An alternate and untested hypothesis is that responses to sudden stops of visual flow are simply due to the convergence of motor and visual inputs and do not arise from the precise coupling between an animal’s actions and the visual stimulus.

## Results

**Figure 1:**
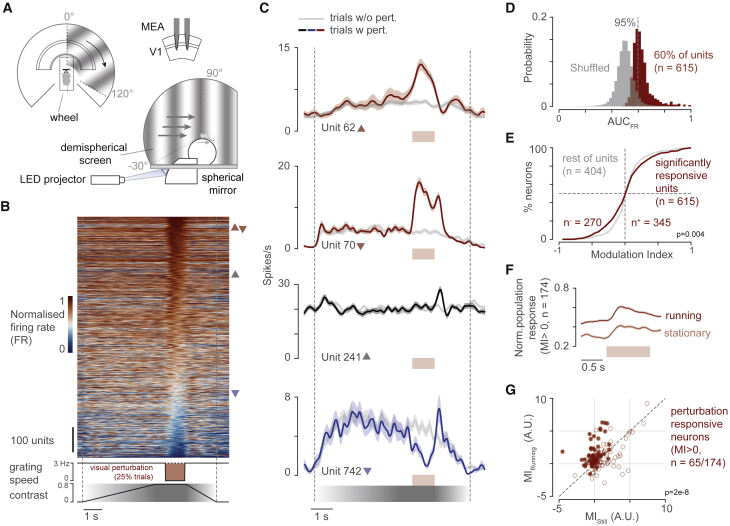
V1 neurons show responses to visual flow perturbations that are stronger during running (A) Top and side view of the recording apparatus. Top right: schematic of the multi-electrode array (MEA) silicon probe and recording area. (B) Top: normalized mean response of all recorded units (n = 1,019) for trials with perturbation. Firing rate of single units was normalized by their maximum recorded during the session. Units are sorted in descending order based on the mean firing rate during the perturbation period. Bottom: contrast and temporal frequency of the drifting grating for trials with perturbation (temporal frequency, TF = 0 cycles/s). Triangles refer to the position of example units shown in (C). (C) Mean response to trials with perturbation (red/black/blue trace) and without perturbation (gray trace) of four example units. Units 62, 70, and 742 are significantly responsive to perturbation (red and blue traces indicate positive and negative modulation, respectively); the third example is not perturbation responsive. Unit number is consistent with rows in (B). Shaded areas indicate SEM. (D) Probability distribution of the area under the receiving operating characteristic curve of logistic classifier trained on shuffled and recorded data. Dotted line represents the 95 percentile threshold. (E) Probability cumulative distribution of the modulation indices (MIs) of perturbation-responsive units (red) and the other units (gray). p value from two-sample Kolmogorov-Smirnov test. (F and G) Modulation of perturbation responses in running trials versus stationary trials for neurons recorded in sessions in which there were at least 4 trials for each condition (n = 174 neurons). (F) Normalized mean population firing rate activity during perturbation trials in running and stationary conditions. Shaded areas indicate SEM. (G) The modulation of each unit, for each condition, was computed as the sum of all firing rates of the perturbation period minus those evaluated during 1 s preceding the perturbation. Solid dots show units that were significantly modulated by running individually (non-parametric test with significance threshold at p = 0.05, see STAR Methods for details). See also Figures S1 and S2.

To test how changes to visual flow affected neural activity in the V1, we presented gratings drifting at a constant speed in eight directions (0.04 cycles/°, 3 cycles/s, 0°:45°:315°, trial duration = 7.3 s), in the right visual field (covering 120° by 120°, Figure 1A). Mice were head restrained and free to run on a polystyrene wheel. The open-loop stimulus allowed measurements in the absence of explicit sensorimotor expectation and therefore characterizing the individual contribution of motor and visual inputs. For example, the open-loop stimulus allowed us to test different stimulus directions compared to closed-loop in which only front-to-back motion would be viable. On each trial, the contrast of the drifting grating slowly increased from 0 to 0.8 and was then held constant for 1 s, before decreasing back to 0 again. We chose this protocol so that mice did not experience sudden, transient changes of stimulus appearance or speed. On the recording day, however, the drifting grating was suddenly stopped for 1 s in a random 25% of the trials, thus causing an unexpected perturbation of the visual flow (Figure 1B).

The perturbation of purely visual flow was clearly reflected in the activity of neurons recorded in V1 (Figure 1B). We used multi-electrode array silicon probes to record the activity of neurons in the visual cortex. Many individual neurons showed a change in their firing rates during the perturbation (Figures 1B and 1C). To quantify the reliability of each neuron’s perturbation response, we trained a logistic regression classifier to discriminate between perturbation and non-perturbation trials based on features of an individual neuron’s activity, including response amplitude during the visual flow halts (Figures 1C and 1D; see STAR Methods for details). The classifier would discriminate only between the trial types when the perturbation responses are large and reliable. We considered a unit reliable if the classifier performed better than 95% of classifiers trained on shuffled data (Figure 1D), and we found that 60% of neurons (n = 615/1,019) were reliably responsive to visual perturbation. As the classifier could use either positive or negative perturbation responses, we also characterized the sign of the responses by measuring a modulation index (MI), which is the proportional change in firing rate during the perturbation, relative to the pre-perturbation period. We found that most neurons responded to the perturbation by increasing their firing rate (n = 345 of 1,019 versus 270 of 1,019 units that reduced responses; Figure 1E). Perturbation-responsive units were similarly represented in both putative fast and regular spiking classes, so we analyzed them together.

Perturbation-responsive neurons appear to respond as if they encode visuomotor mismatch: activity increases when the visual flow, otherwise coupled to the animal running, is suddenly stopped. We therefore evaluated the effects of running on the perturbation responses, in sessions that had at least 4 trials in both running (speed, ≥2 cm/s) and stationary conditions. Although perturbation responses were present in both stationary and running conditions, they were markedly different, as follows: pre-perturbation activity was higher during running (Figure 1F; p = 0.002, Wilcoxon rank-sum test), and the MI was higher when the animal was running than when it was stationary (n = 65/174 units, p < 10^−7^, Wilcoxon rank-sum test). Therefore, the perturbation responses were larger when animals were running.

Visual perturbation responses were not explained by perturbation-induced changes in running behavior. We quantified changes in running speed in each recording session by using the same metrics as for neural responses, calculating reliability and MIs for speed changes. Mice showed reliable changes in speed following the perturbation in less than one-half the sessions (n = 19/47 sessions with 95 percentile performance; Figures S1A and S1B), and these sessions had a similar fraction of perturbation units as sessions with no running speed changes (35% compared to 34% in other sessions). Importantly, trial-by-trial MIs of neural responses were rarely (n = 8/345 units) correlated with MIs of speed changes (Figures S1C, S1D, and S1E). Therefore, neither the occurrence nor the magnitude of changes in running speed explained perturbation responses.

A crucial feature of the sensorimotor mismatch hypothesis is that perturbation responses will be largest for the motion direction predicted by the animal’s running (front-to-back or naso-temporal flow). Previous reports of responses during sensorimotor mismatch were measured using stimuli moving only along this direction, leaving open the question of whether perturbation responses might also be present for other motion directions. We therefore tested the perturbation response in eight different directions—in each case, the stimulus moved in a particular direction and suddenly stopped during the perturbation period.

**Figure 2:**
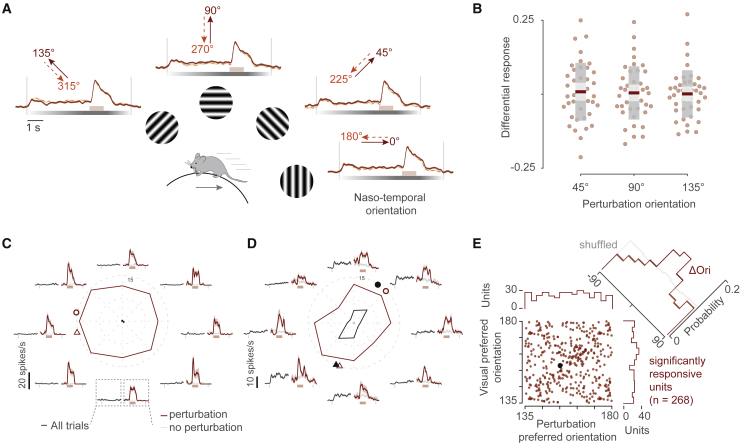
Perturbation responses are not biased to the front-to-back direction and are stronger at the preferred orientation of single neurons (A) Mean normalized population responses of the perturbation-responsive units (only positively modulated) for different grating directions. Dotted lines indicate the start/end of the trial and of the perturbation period. Firing rate of single units was normalized by their maxima. Shaded areas indicate SEM. (B) Perturbation responses to orientation angles for all recording sessions (n = 41) minus responses to naso-temporal orientation. Colors of boxplots indicate mean (red), SEM (light gray), and SD (dark gray). (C and D) Mean responses of two example units for the different grating directions. The central panel of each example shows the mean responses for different grating directions computed during the first 4 s of the trials minus the baseline activity (black line). Red line is the mean firing rate computed during the visual perturbation period (same baseline as the grating stimulus is used to avoid any bias from visual direction/orientation tuning of the unit). Surrounding plots show mean responses for each direction. The responses to the visual stimulus (0–4 s) are averaged over all trials in the direction. Responses to the visual perturbation are averaged over the number of trials for each condition (red, with perturbation; light gray, no perturbation). Circles indicate preferred orientation. Triangles indicate preferred direction. (E) Scatterplot of preferred orientation angle for grating stimulus and visual perturbation. Black data point indicates example unit shown in (D). Only units tuned to orientation are considered (Hotelling’s *T*^2^ test, p ≤ 0.01 n = 268). Marginal distributions above and to the right of the plot show preferred perturbation and visual orientation angles, respectively. The distribution of the difference between the preferred perturbation and visual orientation angle of each neuron (ΔOri) is shown on the top right. Shuffled data are indicated in gray. See also Figure S3.

We found that population perturbation responses were similar in every motion direction tested (Figure 2). For each direction, we measured the population perturbation response as the average normalized response across all the perturbation units within a recording session (n = 47 recordings). The population perturbation responses did not display a preference for any direction (Figure S3B) or orientation (Figure 2B). We next asked if individual neurons showed any preference to the direction of the perturbation. Although some neurons showed no preference for the orientation of the perturbation (Figure 2C), we found some neurons that had a higher response along certain orientations (Figures 2D and 2E). We defined the orientation with the maximal perturbation response as the preferred perturbation orientation. The distribution of preferred perturbation orientations did not show a bias for any particular orientation or direction (Figure 2E; Figure S3C), which is consistent with the lack of bias in the population responses. Therefore, the perturbation responses were not biased in the front-to-back direction as predicted by a sensorimotor mismatch.

The perturbation responses were influenced by the orientation selectivity of the neuron (Figures 2C and 2E). We measured the orientation selectivity in the period before the perturbation onset (pre-perturbation) and found 268/345 perturbation units to be orientation selective (Hotelling’s *T*^2^ test, p < 0.01). Interestingly, the preferred perturbation orientation often matched the preferred orientation of neurons (Figures 2D and 2E). Neurons with significant orientation selectivity showed a perturbation response tuning similar to that obtained with the drifting gratings (Figure 2D and Figure S3D). The distribution of the difference between the preferred visual and perturbation orientation was centered close to zero (0° ± 30° for n = 104/268 neurons, Figure 2E). We did not find a significant relationship with direction preference of the neurons (Figure S3C). These results suggest that the perturbation responses are more influenced by sensory feature selectivity of the neuron than by running direction.

The perturbation responses, although not consistent with trial-by-trial sensorimotor expectations, might be related to expectations built up by prior exposure to the visual stimuli. To test this hypothesis, we recorded from two groups of animals, as follows: one group (experienced mice, n = 7 subjects, 37 recording sessions) was exposed to the open-loop drifting gratings (without perturbations) over 8–13 days, whereas the other group (naïve mice, n = 3 subjects, 10 recording sessions) did not experience any stimuli before the first recording session. We found a similar percentage of perturbation units in both groups (60% for naive and 58% for experienced mice; Figure S2), suggesting that prior exposure did not influence the occurrence of perturbation responses.

**Figure 3:**
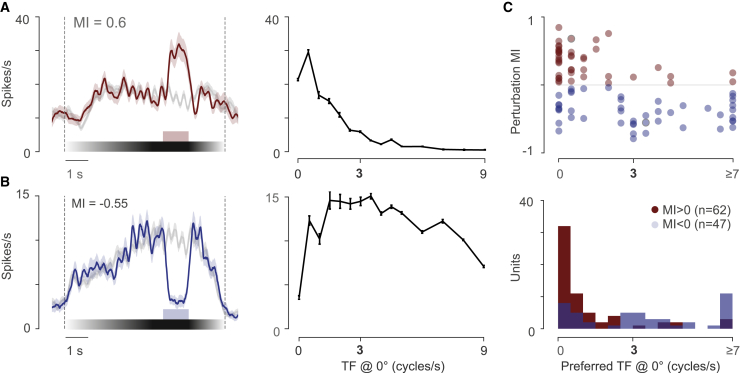
Temporal frequency tuning can explain perturbation responses (A) Example neuron positively modulated by the visual perturbation. Left: mean response to perturbation trials (red trace) and non-perturbation trials (black trace). Shaded area indicate SEM. MI stands for modulation index of perturbation response. Right: temporal frequency (TF) tuning. Error areas indicate SEM. (B) Like in (A) but with example neuron negatively modulated by the visual perturbation. (C) Top: perturbation MI as a function of the preferred speed for all units. Circled data points are units shown on (A) and (B). Bottom: distribution of preferred TF for positively (red, n = 62) and negatively (blue, n = 47) modulated perturbation-responsive units. All preferred speeds greater or equal to 7 cycles/s are grouped for illustration purposes. See also Figures S4 and S5.

What properties of a neuron determine whether or not it is perturbation responsive? Although expectation was a potential hypothesis, our evidence did not support it, and instead we found a larger role for stimulus orientation. We therefore investigated whether additional stimulus properties predict neural responses to visual flow perturbations. Given that the perturbation responses occurred when the drifting grating was suddenly stopped (or made static), we hypothesized that the positively modulated perturbation units preferred static gratings over drifting gratings. To test this hypothesis, we tested a range of speeds of the grating (different temporal frequencies at a fixed spatial frequency), moving along the naso-temporal (front-to-back) direction (Figure S4A; 3 animals, n = 109 neurons, temporal frequencies tested: 0:0.5:8.5 cycles/s; Figures S4B and S4C). The speed preferences of perturbation units were consistent with our hypothesis (Figure S4H): positively modulated units mostly preferred static gratings, whereas negatively modulated units mostly preferred speeds greater than 3 cycles/s (MI > 0, n = 62; MI < 0, n = 47, Mann-Whitney U test, p = 1.3e-6; Figure 3C). We then considered the possibility that perturbation responses could be explained by direction selectivity. For instance, a grating moving in the opposite or orthogonal direction from that preferred by the neuron might suppress its firing rate. However, we found that the preference for slow speeds was not explained by a preference for other motion directions (Figure S4I). These data suggest that a preference for static or slower moving gratings explains the increases in firing rate observed in response to the visual flow perturbation, irrespective of predictions based on running direction.

## Discussion

We have shown that a significant proportion of neurons of the visual cortex of mice respond to visual flow perturbations during passive viewing of drifting gratings. Although these responses were enhanced when animals were running, the presence or strength of perturbation responses did not depend on the direction of the previously experienced visual flow. Visual perturbation responses were instead better explained by the preferred orientation of the neuron and preferences for static or slowly moving stimuli. This finding suggests that perturbation responses can be explained by visual feature tuning that is enhanced by locomotion and do not require internal monitoring of prediction error relative to self-generated motion.

Neurons responding to visual perturbations are qualitatively similar to sensorimotor mismatch neurons previously described. Specifically, neurons have been classed as sensorimotor mismatch neurons when their responses match the following conditions (Keller et al., 2012): they increase their activity in response to sudden stops of visual flow, and the response magnitude is at least twice as strong when animals run. We have shown here that many visual perturbation neurons can also satisfy these conditions. Using the same criterion, we found 13% of recorded neurons could be classed as sensorimotor mismatch neurons, which is in range of the percentages previously reported (Keller et al., 2012; Liebscher et al., 2016; Zmarz and Keller, 2016; Attinger et al., 2017) (which vary between studies from 5% to 39%).

Our observation that visual perturbation units would be classed as sensorimotor mismatch neurons does not exclude the possibility that there are neurons that are indeed selective to true sensorimotor mismatch, especially in animals that have had an extended experience of closed-loop conditions (Attinger et al., 2017). However, to assess the prevalence of purely sensorimotor mismatch neurons, one would have to discount effects of stimulus properties, also present in open-loop conditions, that explain visual perturbation sensitive units shown here. True visuomotor mismatch neurons should have greater responses to visual perturbations in closed-loop than during playback of the same stimuli while the animals are running, and these responses should be higher in the direction of expected visual flow during locomotion. The visual tuning properties of neurons that respond to sudden stops (perturbation or mismatch) are consistent with typically reported properties of V1. The direction tuning of perturbation responses was not biased in the expected visual flow direction—the front-to-back direction. Instead, we found perturbation responses to be better predicted by the orientation tuning of the neurons. Previous work characterizing visual responses to sudden stops in running animals have only explored responses to front-to-back motion. Neurons responding positively to a sudden stop also prefer slow speeds. That is, they prefer the speed experienced during the sudden stop, rather than that experienced during the visual flow. This simple visual tuning is enhanced during running, a well-known property of visual neurons (Niell and Stryker, 2010; Saleem et al., 2013; Busse et al., 2017).

For a neural network to implement predictive coding, a fraction of neurons need to encode prediction errors (Keller and Mrsic-Flogel, 2018). In the case of visual flow, high prediction error would be represented by high activity levels when an animal is running fast, but experienced visual flow is slower. The sensorimotor mismatch model suggested encoding prediction errors in V1 combines an excitatory efference signal (motor command) and an inhibitory sensory feedback (Attinger et al., 2017; Jordan and Keller, 2020). However, our data show that it is possible that a preference for low visual flow and high running speeds, which are both potentially excitatory inputs, can produce responses consistent with a prediction error signal. Our observations also explain why the distribution of visual speed tuning preferences across the layers of V1 are consistent with the prevalence of neurons responsive to sensorimotor mismatch (Jordan and Keller, 2020): superficial layers that have a larger fraction of mismatch neurons than deeper layers also have a larger fraction of neurons preferring low temporal frequencies (based on the Allen Institute dataset; de Vries et al., 2020; Figure S5). Therefore, encoding of prediction error in the visual cortex can be achieved by the convergence of distributed visual tuning properties with a modulation of responses by running.

Features consistent with the predictive coding hypothesis have been observed in primary sensory areas in responses to the omission of expected stimuli (Fiser et al., 2016; Garrett et al., 2020) and suppression of responses to expected sounds (Rummell et al., 2016). Similarly, internal representations of spatial location predict the responses of upcoming stimuli (Fiser et al., 2016) and modulate the responses to identical sensory stimuli (Saleem et al., 2018; Poo et al., 2020). These responses are often explained as the difference between efference information and the sensory signal, but our results propose a simpler model that may be useful, as follows: the convergence of distributed sensory and motor response codes. Incorporating and accounting for these factors provide a richer testbed for theories of sensory coding during self-generated actions.

## STAR★Methods

### Key resources table

**Table:** 

Reagent or resource	Source	Identifier
Isoflurane	Piramal Critical Care	CAS 26675-46-7

**Experimental models: organisms/strains**		

Mouse: C57BL6/J	Charles River Laboratories	RRID:IMSR_JAX:000664

**Software and algorithms**		

MATLAB 2019a	Mathworks	https://www.mathworks.com/
OpenEphys	Siegle et al., 2017	https://open-ephys.org/
Bonsai 4.3	Lopes et al., 2015	https://bonsai-rx.org/
BonVision	Lopes et al., 2021	https://bonvision.github.io/info/Home/#

### Resource availability

#### Lead contact

Further information and requests for resources and reagents should be directed to and will be fulfilled by the Lead Contact, Aman Saleem (aman.saleem@ucl.ac.uk).

#### Materials availability

This study did not generate new unique reagents. Commercially available reagents are indicated in the Key resources table.

### Experimental model and subject details

All experiments were performed in accordance with the Animals (Scientific Procedures) Act 1986 (United Kingdom) and Home Office (United Kingdom) approved project and personal licenses. The mice (n = 10 C57BL6 wild-type, 7 females and 3 males, age 16-24 weeks) were housed in groups of maximum five under a 12-hour light/dark cycle, with free access to food and water. All electrophysiological recordings were carried out during the dark phase of the cycle.

### Method details

#### Surgery

Mice were implanted with a custom-built stainless-steel metal plate on the skull under isoflurane anesthesia, and allowed to recover for seven days with analgesia. The area above the left visual cortex was kept accessible for electrophysiological recordings. Seven days following the surgery mice underwent the first habituation session. Following the habitation period (one session per day, 8-13 days), a craniotomy was performed over V1, centered at 2 mm lateral to sagittal midline and 0.5 mm anterior to lambda). The dura was left intact to preserve the brain tissue, and maximize recording time. Mice were allowed to recover for 4-24 hours before the first recording session. Multiple recording sessions were performed on each animal (one per day, n = 37 recordings, min 2, max 9). Three animals (naive) were exposed to only gray screen during the habituation sessions (5-10 sessions) and were exposed to the visual stimulus for the first time on the day of the first recording session.

#### Visual Stimulus

The display apparatus was similar to those used in previous studies (Schmidt-Hieber and Häusser, 2013; Muzzu et al., 2018). Mice were head-fixed on a polystyrene wheel (Saleem et al., 2018) (radius 10 cm), with their heads positioned in the geometric center of a truncated spherical screen onto which we projected the visual stimulus. The visual stimulus was centered at +60° azimuth and +30° elevation and had a span of 120° azimuth and 120° elevation.

The visual stimulus was designed using BonVision (Lopes et al., 2021), an open-source visual environment generator based on graphical programming language of the Bonsai framework (Lopes et al., 2015). A session was structured in trials during which a drifting sinusoidal grating was presented for approximately 7.3 s (sinusoidal grating with spatial frequency of 0.04 cycles/° and temporal frequency of 3 cycles/s). The grating was made visible by increasing the contrast from 0 to 0.8 in the first 240 frames of the trial. Contrast remained at 0.8 for the following 100 frames and then decreased to zero in 100 frames. Total trial duration was 440 frames at 60 Hz frame rate, i.e., 7.3 s. The direction of the grating was randomly picked between eight directions: 0°:45°:315°. Each grating direction was shown at least twenty times. For each combination of directions a trial was shown with zero contrast. The inter-trial interval varied randomly between 1 to 2.5 s. The firing rate during this inter-trial interval and the zero-contrast trial was used as a baseline. Habituation sessions for naive mice were run with a gray screen.

The visual perturbation was presented in a random 25% of the trials during the recording sessions. The perturbation onset happened between 30 and 40 frames after the contrast had reached its highest value (0.8), and the perturbation lasted between 60 and 70 frames.

### Quantification and statistical analysis

#### Spike sorting and clustering

To record the neural activity we used multi electrode array silicon probes with two shanks and 32 channels (ASSY-37 E-1, Cambridge Neurotech Ltd, Cambridge, UK). Electrophysiology data was acquired using an OpenEphys acquisition board (Siegle et al., 2017).

The electrophysiological data from each session were processed using Kilosort version 1 (Pachitariu et al., 2016). Spike times were synchronized with the behavioral data by aligning the signal of a photodiode that detected the visual stimuli transitions (PDA25K2, Thorlabs, Inc., USA). They were then analyzed conjointly in MATLAB R2019a. Firing rate was sampled at 60 Hz and smoothed with a 300-ms Gaussian filter.

#### Logistic regression classifier

To estimate the reliability of the responses to the visual perturbation we trained a trial classifier based on a logistic regression model (MATLAB function *fitglm*). Neural responses of single units were normalized by their maximum values recorded during the entire session before being processed. We used six parameters from each trial as inputs for the model:

1) the ratio of mean firing rate during the perturbation period ($R_{pert}$) and during the second preceding it ($R_{pre-pert}$): $\frac{R_{pert}}{R_{pre-pert}}$

2) the difference between them: $R_{pert}-\;R_{pre-pert}$

3) their sum across the whole period: $\sum R_{pre-pert}+\sum R_{pert}$

4) the summed firing rates during the perturbation period: $\sum R_{pert}$

5) a depth of modulation index:

(Equation 1) $$DM=\;\frac{R_{pert}-R_{pre-pert}}{R_{pert}+R_{pre-pert}}.$$

6) a response modulation index:

(Equation 2) $$MI=\;\frac{R_{pert}-R_{pre-pert}}{R_{pre-pert}}$$

The output of the classifier was the identity of the trial: 1 for perturbation or 0 for non-perturbation trial. We then computed the receiving operating characteristic curve (true positive rate VS negative positive rate) and its area under this curve (AUC) was used as reliability parameter. We then shuffled the trial type 1000 times and rerun the above steps to evaluate the statistical significance of this metric. If the AUC of each unit was greater than 95% (p < 0.05) of the AUC’s computed from all shuffled data, we considered the unit as perturbation responsive.

#### Modulation index

We quantified the magnitude of the perturbation response and identified whether a unit increased (MI > 0) or decreased (MI < 0) its firing rate during the perturbation, using the modulation index as defined in Equation 2.

#### Responses during running

To evaluate the effects of running on perturbation responses we divided the perturbation trials in two groups based on the running state of the mice. If the mouse ran at speeds greater than 2 cm/s during the perturbation period and the second preceding it, we consider it a running trial. Other trials are considered stationary trials. As the animals were free to run or stand still as they wished, we often found an imbalance in the number of stationary and running trials. To better compare the two conditions, we selected only sessions that had at least 4 trials per condition. This allowed us to compare the activity of 174 neurons from 22 sessions. We measured the modulation of the running and stationary trials as the area under the response curve during the perturbation period (analytically is the sum of all firing rates) minus the area under curve evaluated during the pre-perturbation (1 s).

(Equation 3) $$MI_{run|still}=\;\overset{t_{pert-OFF}}{\underset{t_{pert-ON}}{\sum }}{\bar{R}}_{run|still}\left(t\right)-\overset{t_{pert-ON}}{\underset{t_{pert-ON}-1s}{\sum }}{\bar{R}}_{run|still}\left(t\right)$$

where ${\bar{R}}_{run|still}\left(t\right)$ is the average of the mean instantaneous firing rates of either the run or still perturbation trials. To compensate for biases due to smaller number of trials for either condition, we compared the activity of 20 trials per condition by re-sampling 1000 times with replacement. An individual neuron was classified as significantly modulated by running if both the peaks and the mean responses computed for running trials $R_{run}\left(t\right)$ were larger than those computed for still trials $R_{still}\left(t\right)$ in 950/1000 of the cases (5% significance level).

#### Orientation and direction tuning

We estimated the mean responses to the drifting grating at different angles by measuring the mean firing rate in the first 3.5 s from the onset of the stimulus and subtracting the pre-stimulus mean firing rate. Similarly, the neural response for different perturbation directions were computed as the mean firing rate during such period, minus the pre-stimulus (inter-trial period) mean firing rate. We then estimated the preferred orientation and direction tuning of the neurons for the drifting grating using standard methods (Mazurek et al., 2014). Briefly, responses to various directions and orientations were evaluated in either a direction or orientation vector space, and we evaluated the normalized length of the vector sum as a measure for direction and orientation selectivity:

(Equation 4) $$L_{ori}=\;\left|\frac{\sum_{k}R\left(\theta_{k}\right)e^{\left(2i\theta_{k}\right)}}{\sum_{k}R\left(\theta_{k}\right)}\right|$$

where $R\left(\theta_{k}\right)$ is the response to angle $\theta_{k}$

(Equation 5) $$L_{dir}=\;\left|\frac{\sum_{k}R\left(\theta_{k}\right)e^{\left(i\theta_{k}\right)}}{\sum_{k}R\left(\theta_{k}\right)}\right|$$

The preferred orientation or direction of a unit were estimated from the angles of the vectors in the numerator of Equation 4 and Equation 5, respectively. The significance of orientation tuning was computed using Hotelling’s $T^{2}$-test, which is a multivariate generalization of the Student’s T-test. The significance of direction tuning was computed using “direction dot product” test. Significance level was set at 99%. For perturbation responses, we used the direction/orientation with the maximal responses, as the number of trials for each direction was too small to implement the aforementioned method.

#### Temporal frequency tuning

To estimate the temporal frequency tuning we used stimuli with similar specifications to those used for the visual perturbation stimuli (sinusoidal grating only at 0° direction and SF = 0.04 cycles/°, contrast = 0.8, Figure S4). This was presented only to naive mice. Each trial commenced with a grating moving at either 1.5, 3, or 6 cycles/s. After one second (60 frames) there was a period of acceleration, where the temporal frequency linearly increased or decreased at a random rate between seven possible values (−3:1:3 cycles/s^2^) for 0.5 or 1 s (30 or 60 frames). At the end of the acceleration periods the grating speed was kept constant for 1.5-2 s (90-120 frames) followed by a gray inter-trial interval of 1 to 2.5 s. Twenty-one combinations of initial temporal frequencies and acceleration rates were presented twenty times each. The mean firing rates of the final second of all trials with a specific temporal frequency were used to calculate the speed tuning curve for the perturbation-responsive neurons (n = 109). Preferred temporal frequency was the temporal frequency with the maximal response.

#### Classifying regular and fast spiking units

To estimate whether the units were regular or fast spiking, we used an automated classification algorithm (MATLAB function *kmeans*) applied onto the first three principal components (> 90% variance explained) of the mean spike waveforms of all units. The two main clusters had a mean trough-to-peak duration of 0.83 ± 0.15 ms (n = 684 units, mean ± std) and 0.37 ± 0.12 ms (n = 255 units), which we considered putative regular and fast spiking units respectively. The trough-to-peak duration was measured as the delay between the trough of the spike and the successive local maximum. This measure is equivalent to the width of the action potential waveform at half maximum (McCormick et al., 1985). Perturbation-responsive neurons made up 67% of fast spiking and 58% of regular spiking units.

## Data Availability

New code is available on GitHub here: https://github.com/SaleemLab/MuzzuSaleem_2021. Data collected in this study is available upon request. Any additional information required to reanalyze the data reported in this paper is available from the lead contact upon request.

## Supplemental information

Document S1. Figures S1–S5

Document S2. Article plus supplemental information
