# Feature selectivity can explain mismatch signals in mouse visual cortex

**DOI:** 10.1016/j.celrep.2022.110413

**Published:** 2022-02-15

**Authors:** Tomaso Muzzu, Aman B. Saleem

## Main text

(Cell Reports *37*, 109772; October 5, 2021)

In the originally published version of this article, the tick labels for orientation angles in Figure 2E were missing values. A similar error was also present in Figure S3C. The original and corrected Figures 2E and S3C are shown below, and the corrected figures now appear with the article online. This error does not affect the data presentation or the conclusion of the paper.

The authors regret this error.

**Figure:**
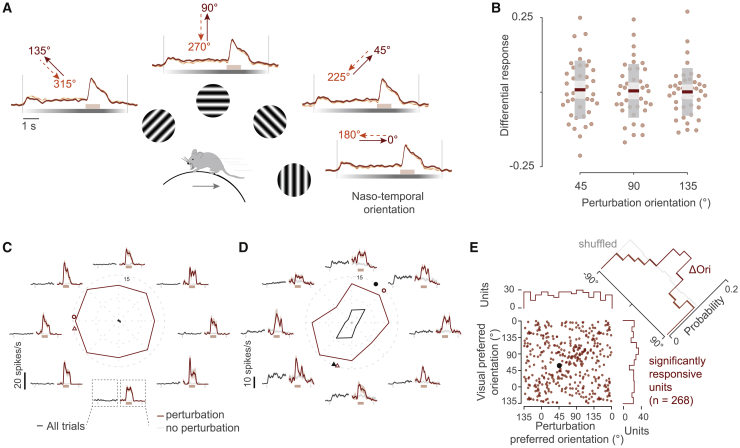
Figure 2. Perturbation responses are not biased to the front-to-back direction and are stronger at the preferred orientation of single neurons (corrected)

**Figure:**
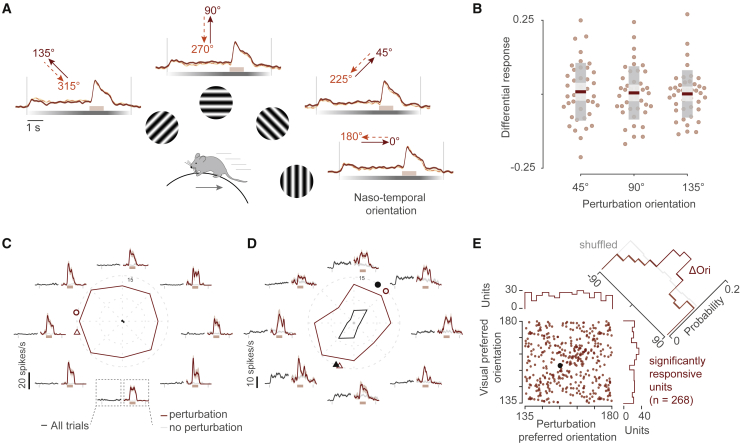
Figure 2. Perturbation responses are not biased to the front-to-back direction and are stronger at the preferred orientation of single neurons (original)

**Figure:**
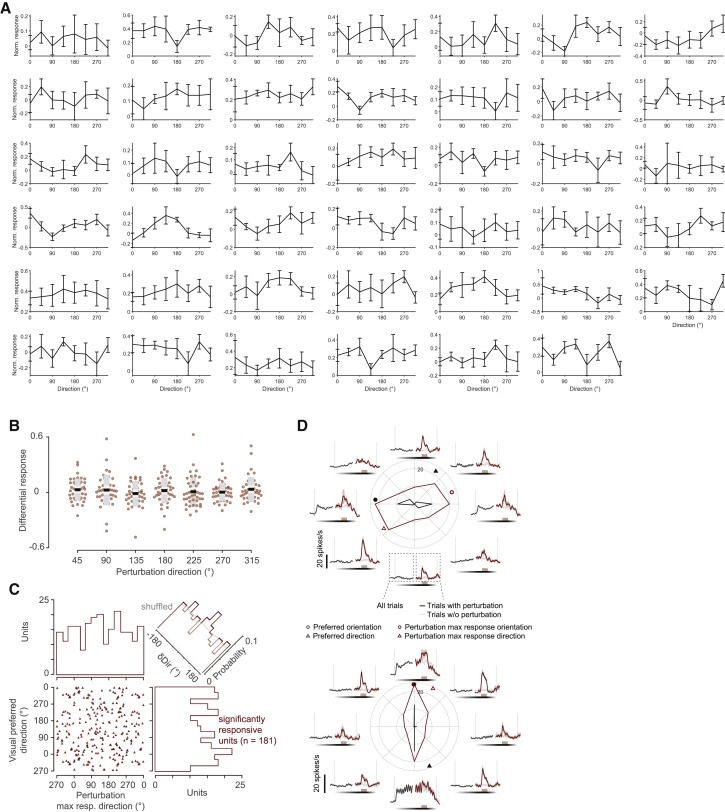
Figure S3. Population responses are not affected by visual flow direction, related to Figure 2 (corrected)

**Figure:**
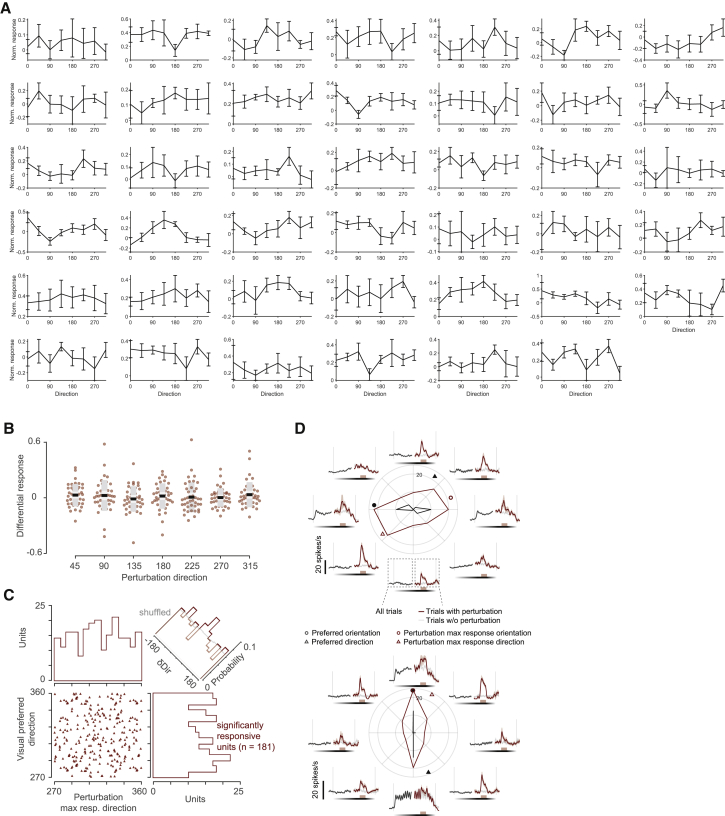
Figure S3. Population responses are not affected by visual flow direction, related to Figure 2 (original)

