# Supplementary material for: Feature selectivity can explain mismatch signals in mouse visual cortex
Source: Cell Rep. 2021 Oct 5;37(1):109772. doi: 10.1016/j.celrep.2021.109772 (PMC8655498; doi:10.1016/j.celrep.2021.109772)
Supplement: Document S1. Figures S1–S5 [file mmc1.pdf]

Cell Reports, Volume *37*

Supplemental information

Feature selectivity can explain mismatch signals in mouse visual cortex

Tomaso Muzzu and Aman B. Saleem

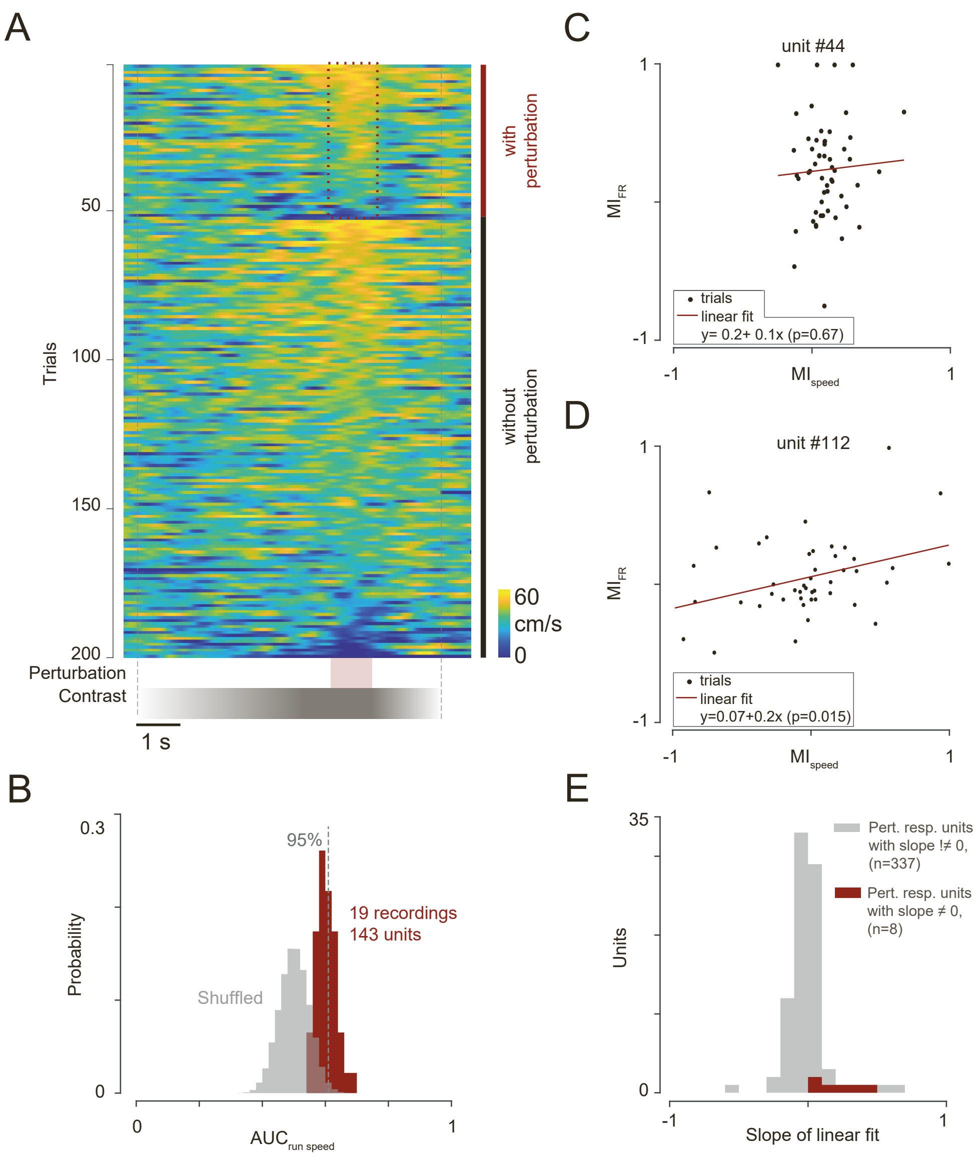

Figure S1, related to Figure 1. Visual perturbation responses are not explained by perturbation-induced changes in running behaviour.

A, Top: normalised running speed during every trial of an example session. Bottom: contrast and visual flow perturbation period (TF = 3 → 0 Hz). **B**, Probability distribution of the area under the receiving operating characteristic curve of the logistic classifier trained with shuffled and actual running speed data. **C**, **D**, scatter plot of modulation indexes for firing rate (MI_{FR}) and running speed (MI_{speed}) for two example units. Modulation is computed as the difference between the mean firing rate (or speed) during the perturbation and the pre-perturbation period, and normalised by the second term. The linear fit (red line) measures the correlation between the two modulations. The *p*-value indicates the *t*-statistic of the hypothesis test that the slope is significantly different from zero or not. **E**, Distribution of the slope of the linear fits for all positively modulated perturbation responsive units. Significance is measured at the 1% significance level.

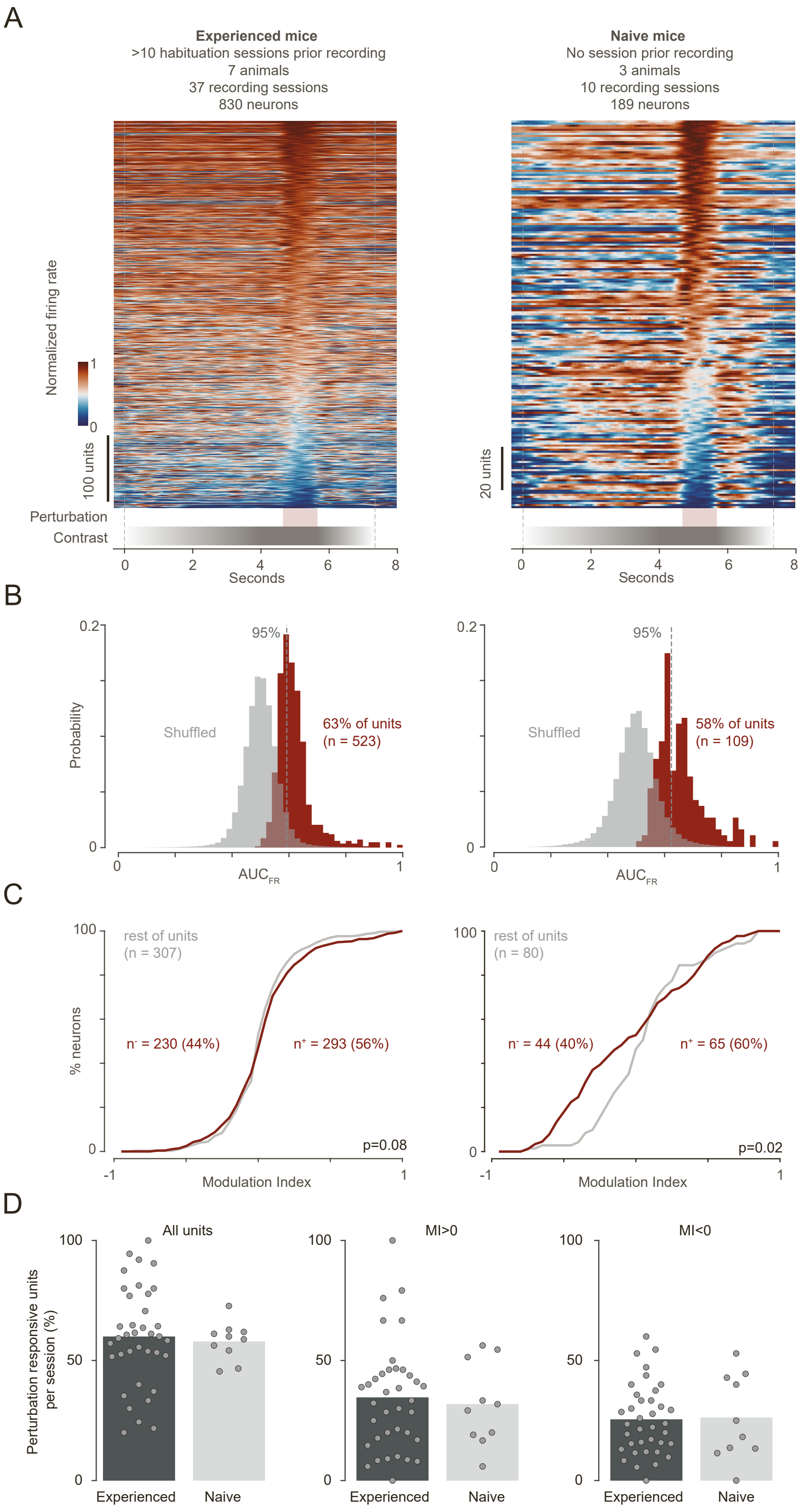

Figure S2, related to Figure 1. Perturbation responses are also present in naïve animals

A, Top: normalised mean response of all recorded units (n=189) for trials with perturbation. Bottom: and visual flow perturbation period (TF=3 → 0 Hz). Left: Experienced mice with at least 8 habituation sessions. Right: Naïve mice with no experience of visual stimulation. Same order applies to other panels. **B**, Probability distribution of the area under the receiving operating characteristic curve of the logistic classifier trained with shuffled and recorded neural data. **C,** Probability cumulative distribution of the modulation indexes (MI) of perturbation responsive units (red) and the other units (gray). P value for two-sample Kolmogorov-Smirnov test. **D,** Breakout of percentages of perturbation responsive units per session for experienced (n=37) and naïve mice (n = 10).

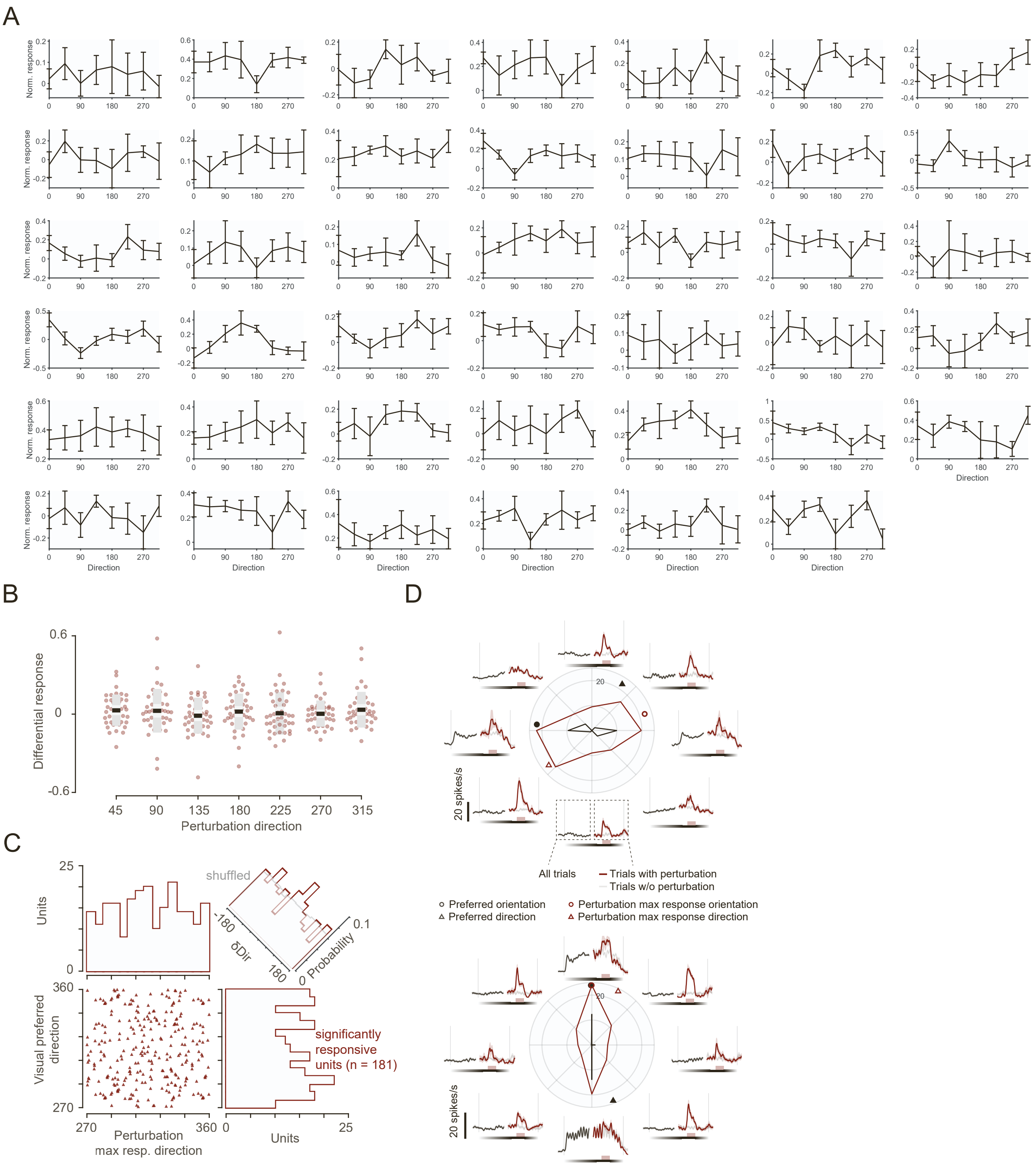

Figure S3, related to Figure 2. Population responses are not affected by visual flow direction

A, Normalised perturbation responses of individual session populations (n>1) for all directions tested (0:45:315°). Error bars indicate SEM. **B,** Perturbation responses for all neuron ensembles of units recorded in a session (n=41, number of units per session ranges between 2 and 24). Colours of box plots indicate mean (black), SEM (light gray), STD (dark grey). **C**, Scatter plot of preferred direction angle for grating stimulus and visual perturbation. Only units tuned to any grating direction are shown (Hotelling's t-squared test, p<=0.01 n=181). Marginal distributions above and right to the scatter plot show preferred perturbation and visual direction angles respectively. The distribution of the difference between the preferred perturbation and visual direction angle of each neuron (δDir) is shown on the top right in red. Shuffled data are indicated in gray. **D**, Mean responses of two example units for the different grating directions. For details please refer to Figure 2 C and D.

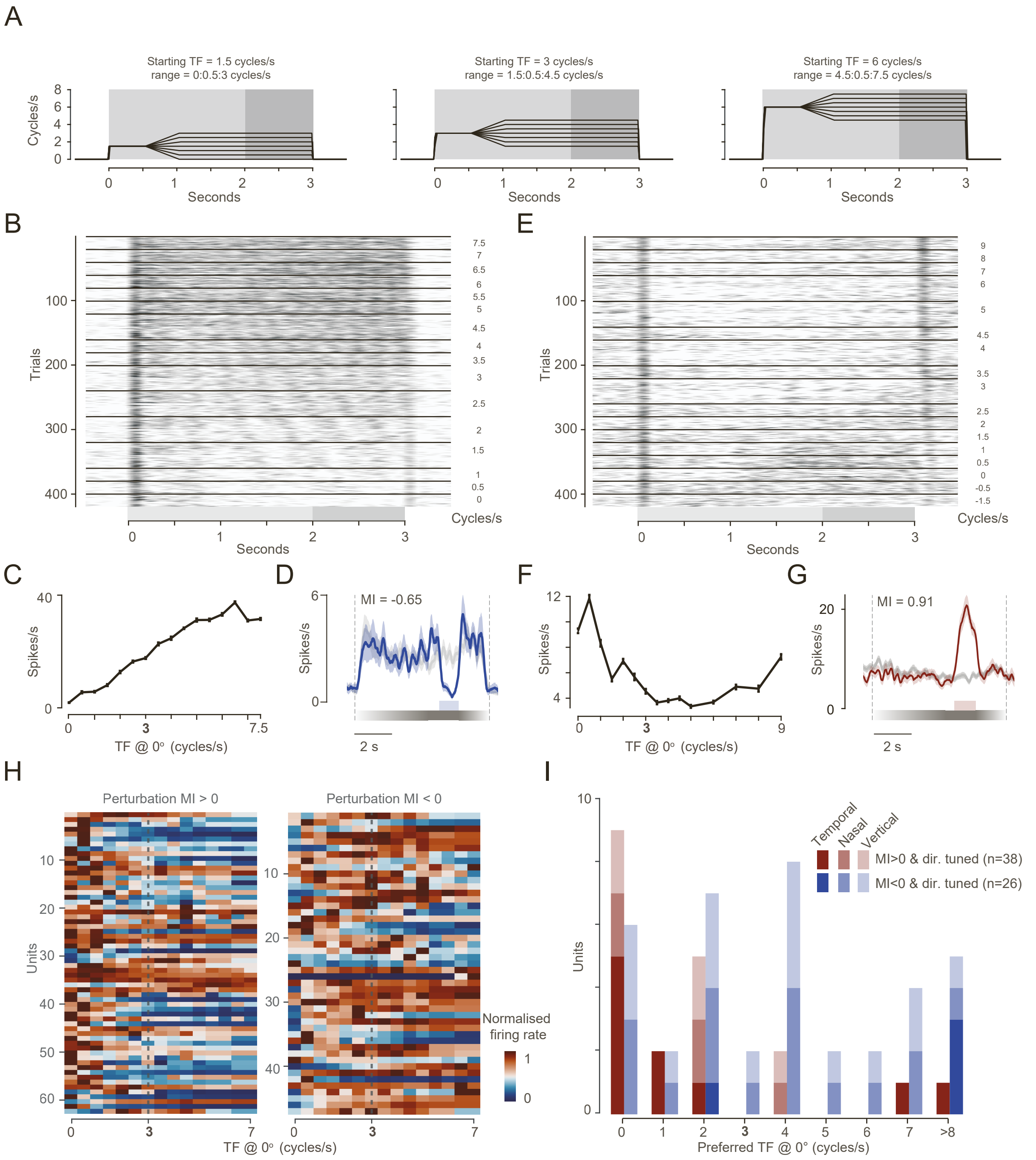

Figure S4, related to Figure 3. Temporal frequency tuning of perturbation responsive units

A, Temporal frequency profiles shown during trial (0 → 3s, grey area). We used 3 starting TF's: 1.5, 3, and 6 cycles/s. After 0.5 s, this was increased or decreased at a random rate -3:1:3 $cycles/s^2$ for 0.5 seconds and then kept constant (see black traces). To compensate for onset transient responses, the temporal frequency tuning was evaluated in the last second of the trial (dark grey area). **B, C, D,** example neuron negatively modulated by the visual perturbation: **C** is the tuning curve of spiking activity during all trials for all temporal frequencies tested; error bars indicate SEM. Mean response to perturbation (red trace) and non-perturbation trials (black trace) is shown in d. Shaded areas indicate SEM. **E, F, G,** like B, C, D but with example neuron positively modulated by the visual perturbation. Please not that in this session, the temporal frequency changed for 1 second instead of 0.5 second, as shown in A. Hence TF values reached higher (and lower) values. We only considered TF's ≥ 0. **H,** Heat map of speed tuning curves computed along the temporal direction for the perturbation responsive units. Dotted line indicates the temporal frequency used in the main stimulus (3 cycles/s) before the perturbation. Left heat map shows larger blue area for speeds >3 cycles/s compared to right indicating a preference for low temporal frequencies of positively modulated perturbation responsive units. **I**. Distribution of preferred temporal frequencies for neurons responsive to visual perturbation (MI>0 in red and MI<0 in blue) and significantly direction tuned (direction dot product test p<0.01). Preferred directions are grouped according to the closest temporal, nasal, and vertical (superior and inferior) axes (angle difference <45°).

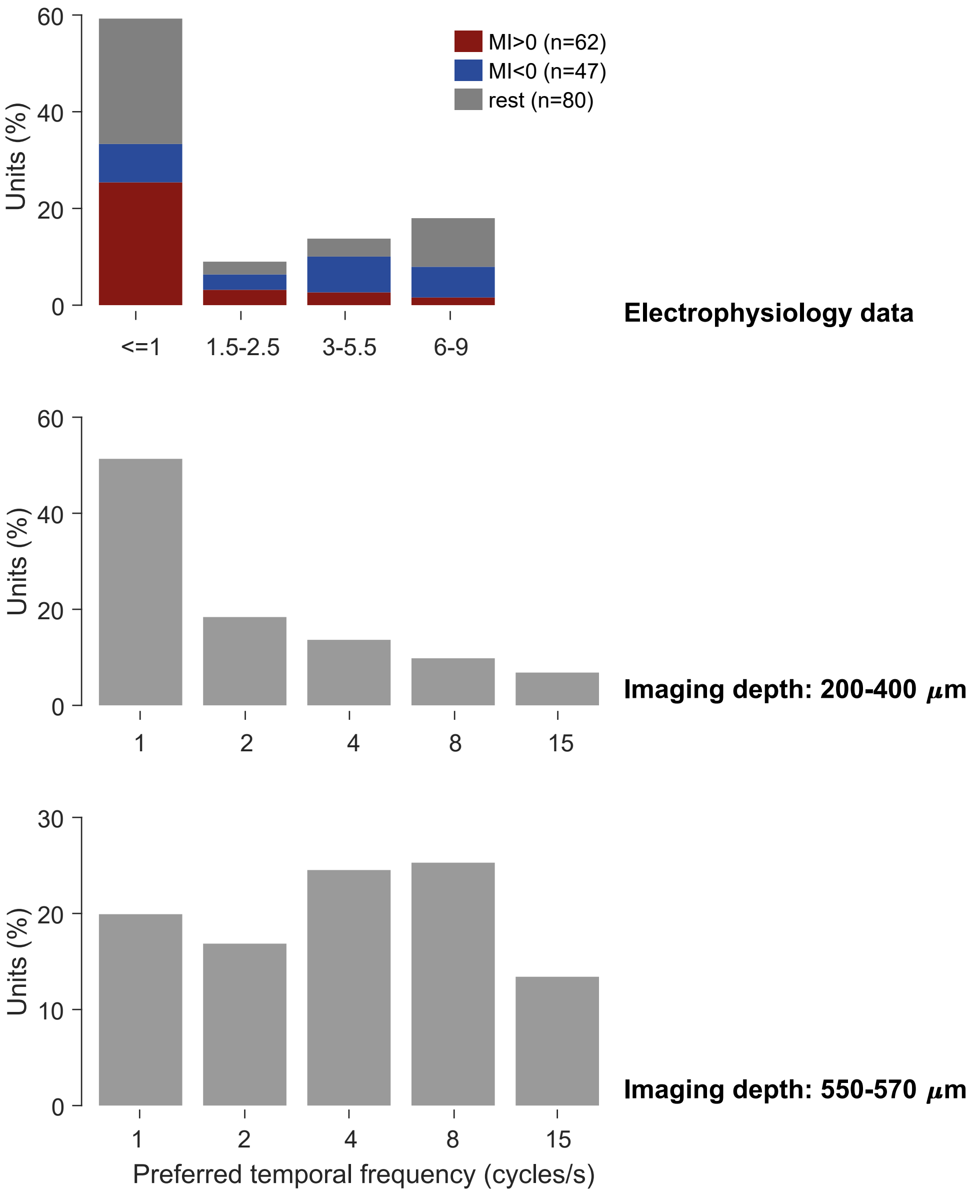

Figure S5, related to Figure 3. Comparison of preferred temporal frequencies with data from Allen Brain Institute.

Distribution of the preferred temporal frequencies for neurons of our dataset (top), Allen Brain Institute for imaging depths of the mouse visual cortex between 200 and 400 μm (middle, n=9059), and for depths of 500-570 μm (bottom, n=261). Data retrieved from the online data portal (url: http://observatory.brain-map.org/visualcoding). Neurons shown here are significantly responsive to drifting grating (p<0.01, n=9059/15619 for superficial layers and n =261/573 for deeper imaging depths.)
